# Hemodynamic Response Alteration As a Function of Task Complexity and Expertise—An fNIRS Study in Jugglers

**DOI:** 10.3389/fnhum.2016.00126

**Published:** 2016-03-30

**Authors:** Daniel Carius, Christian Andrä, Martina Clauß, Patrick Ragert, Michael Bunk, Jan Mehnert

**Affiliations:** ^1^Institute for General Kinesiology and Exercise Science, University of LeipzigLeipzig, Germany; ^2^Department of Sport Science, Martin Luther University of Halle-WittenbergHalle, Germany; ^3^Department of School Sport, Institute of Sport Psychology and Sport Pedagogy, University of LeipzigLeipzig, Germany; ^4^Institute of General Kinesiology and Athletics Training, University of LeipzigLeipzig, Germany; ^5^Department of Neurology, Max Planck Institute for Human Cognitive and Brain SciencesLeipzig, Germany; ^6^Institute for Applied Training ScienceLeipzig, Germany; ^7^Day Clinic for Cognitive Neurology, University Hospital LeipzigLeipzig, Germany

**Keywords:** neuroplasticity, near-infrared spectroscopy, task difficulty, long-term training, juggling

## Abstract

Detailed knowledge about online brain processing during the execution of complex motor tasks with a high motion range still remains elusive. The aim of the present study was to investigate the hemodynamic responses within sensorimotor networks as well as in visual motion area during the execution of a complex visuomotor task such as juggling. More specifically, we were interested in how far the hemodynamic response as measured with functional near infrared spectroscopy (fNIRS) adapts as a function of task complexity and the level of the juggling expertise. We asked expert jugglers to perform different juggling tasks with different levels of complexity such as a 2-ball juggling, 3- and 5-ball juggling cascades. We here demonstrate that expert jugglers show an altered neurovascular response with increasing task complexity, since a 5-ball juggling cascade showed enhanced hemodynamic responses for oxygenated hemoglobin as compared to less complex tasks such as a 3- or 2-ball juggling pattern. Moreover, correlations between the hemodynamic response and the level of the juggling expertise during the 5-ball juggling cascade, acquired by cinematographic video analysis, revealed only a non-significant trend in primary motor cortex, indicating that a higher level of expertise might be associated with lower hemodynamic responses.

## Introduction

Measuring online brain processing during the execution of complex motor tasks with a large motion range still remains challenging. Even though specific non-invasive brain imaging techniques such as electroencephalography (EEG) and brain stimulation techniques (transcranial magnetic stimulation, TMS) have the capacity to investigate brain activity directly, these techniques are highly affected by movement artifacts especially when complex movements with a high motion range are executed (Gramann et al., 2011; Ayaz et al., 2013; Mehta and Parasuraman, 2013). An interesting alternative method for investigating complex movements more thoroughly is functional near-infrared spectroscopy (fNIRS), which similar to functional magnetic resonance imaging (fMRI) measures changes in blood oxygenation (Obrig and Villringer, 2003). FNIRS has been repeatedly shown to be quite robust against movement artifacts not only in simple (Toronov et al., 2000; Cannestra et al., 2003; Franceschini et al., 2003; Kuboyama et al., 2005; Akiyama et al., 2006; Shibuya and Kuboyama, 2007, 2010; Holper et al., 2009; Nambu et al., 2009; Shibusawa et al., 2009; Koch et al., 2010; Mehnert, 2012; Lu et al., 2013; Mehnert et al., 2013) but also in complex motor tasks such as walking and cycling (Miyai et al., 2001; Suzuki et al., 2004, 2008; Perrey, 2008; Harada et al., 2009; Subudhi et al., 2009; Doi et al., 2013; Rupp et al., 2013; Koenraadt et al., 2014; Piper et al., 2014; Tempest et al., 2014; Oussaidene et al., 2015; Nishiyori et al., 2016).

The aim of the present study was to investigate the hemodynamic response within sensorimotor networks [bilateral primary motor cortex (M1), somatosensory cortex (S1) and premotor cortex (PMC)] as well as in visual motion area (MT, V5) during the execution of a complex visuomotor task such as juggling. More specifically, we were interested in how far the hemodynamic response is altered by task complexity. Based on previous findings, higher task complexity in at least simple motor tasks seems to be related with altered hemodynamic response function (Holper et al., 2009). In the present study, we aimed at differentiating between task complexity and altered brain response during the execution of a unilateral 2-ball juggling pattern (right and left hand separately), bilateral 3- and 5-ball cascades.

Furthermore, we were interested in the amount of change of the hemodynamic response during juggling as a function of juggling expertise, an index for the individual learning stage. Short-term learning (over weeks) of simple juggling patterns is associated with structural cerebral changes (Draganski et al., 2004; Boyke et al., 2008; Driemeyer et al., 2008; Scholz et al., 2009). Further studies considering different complex motor tasks report similar findings for structural plasticity associated with short-term motor learning (Taubert et al., 2010; Gryga et al., 2012). There is also strong evidence from studies using fNIRS (Hatakenaka et al., 2007; Ikegami and Taga, 2008; Leff et al., 2008; Gentili et al., 2013) that short-term learning is associated with functional cerebral changes. Since structural MRI studies provided first evidence that the individual juggling expertise (long-term motor learning over years) is associated with larger gray matter density in MT/V5 (Gerber et al., 2014), we also investigated if a similar relationship could be observed in experienced jugglers on a functional level.

In summary, we hypothesized, according to previous reports on simple motor tasks (Holper et al., 2009), that the hemodynamic response in sensorimotor networks as well as visual motion areas increases as a function of task complexity. Since this is the first study investigating neurovascular effects during the execution of a complex visuomotor task (juggling), we obviously cannot make direct inferences about the regional specific modulatory effects in sensorimotor and/ or visual motion areas. Apart from this limitation, we further hypothesized that task-complexity dependent hemodynamic response alterations are reliant on the individual juggling expertise. More specifically, highly experienced jugglers should show less neurovascular alterations in highly complex juggling tasks (e.g., 5-ball cascade) as compared to less experienced peers.

## Materials and methods

### Subjects

Twenty-three healthy male jugglers (2 left-handed) were enrolled in the present study. Jugglers had to fulfill the following criteria in order to be eligible for the study: Participants should be able to maintain a bimanual 5-ball cascade for at least 20 s in eight consecutive trials. Those who fulfilled this criterion were regarded as expert jugglers. Apart from this, all participants had to be free of any neurological and/or psychological disease. Three participants were not included in the final analyses since they were not able to maintain the requested 5-ball cascade. Furthermore, five participants had to be removed due to poor fNIRS signal quality. Hence, a total number of 15 expert jugglers were finally analyzed in the present study. The average age of the 15 expert jugglers was 26.3 ± 5.2 years (ranging from 17 to 38 years). We used a questionnaire regarding their level of expertise (e.g., hours of training/week and years of juggling experience), which is similar to Gerber et al. (2014). Handedness was acquired by the Edinburgh handedness questionnaire (Oldfield, 1971).

### Experimental procedure

Participants filled out the Edinburgh questionnaire and a questionnaire on their level of expertise during the preparation of the fNIRS setup. Subsequently, participants completed a 5 min standardized warm-up session to familiarize themselves with specific juggling balls (SIL-X balls, diameter 78 mm, mass 150 g). Cinematographic and hemodynamic measurements (fNIRS) were performed during the execution of a unimanual 2-ball juggling pattern with either the left or right hand, a bimanual 3-ball and 5-ball cascade. Since task complexity during juggling is associated with higher movement speed, which may influence hemodynamic responses (Obrig et al., 1996), two control tasks were additionally tested. Here, participants had to move their left and right arm in alternating order with a frequency of 1–2 Hz while holding juggling balls in their hands (which was controlled by a metronome).

**Figure 1:**
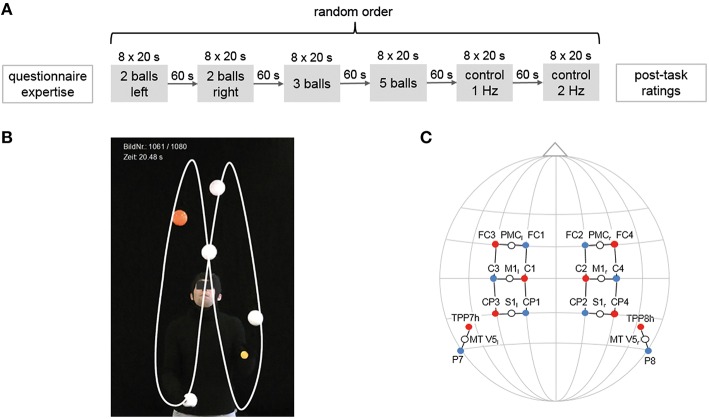
**Experimental design, cinematographic setup and locations of the optode positions. (A)** Experimental design. Participants performed four juggling tasks and two control tasks in random order for 8 × 20 s with 20–25 s rest in between. **(B)** Image section for cinematographic analysis with black backdrop, clothes and white balls. **(C)** Locations of the optode positions (10–20 standard EEG system, fNIRS sources are marked in red, detectors in blue) and measurement channels (white circles) with underlying cortical areas.

During each of the afore mentioned tasks, participants performed the specific juggling task in a block design for 8 × 20 s with 20–25 s rest in between. Between each juggling task, there was a rest period of ~60 s. The order of juggling tasks was randomized for each individual (see Figure 1A). At the end of the experiment, participants rated their perceived level of complexity of each task on a scale from 1 to 10. Additionally, as a qualitative measure, an expert rater ranked the participants (1 being the best, 15 the weakest), by judging their performance (i.e., number of balls and clubs and variability with the execution of the 5-ball cascade; similar to Gerber et al., 2014). This ranking is presented in Table 1.

**Table 1:** **Demographic and behavioral data**.

**Jugglers (sorted by ranking)**	**Age**	**Handed ness**	**Max. number juggled**		**Hours of training (h/week)**	**Years of juggling**	**Ranking order as rated by an expert rater: 1-15**
			**Balls**	**Clubs**			**1 = best juggler; 15 = weakest juggler**
Juggler 1	18	R	9	7	12	5	1
Juggler 2	28	R	9	5	4	16	2
Juggler 3	24	R	7	5	2	10	3
Juggler 4	31	R	7	5	6	16	4
Juggler 5	23	R	8	4	10	2.5	5
Juggler 6	28	R	6	5	12	14	6^*^
Juggler 7	25	L	8	6	1	13	6^*^
Juggler 8	18	L	7	5	8	6	6^*^
Juggler 9	23	RL	7	5	6	10	9^*^
Juggler 10	33	R	8	5	6	11	9^*^
Juggler 11	25	R	7	4	3	5.5	9^*^
Juggler 12	38	R	5	5	1	18	12
Juggler 13	30	R	7	5	0	12	13^*^
Juggler 14	27	R	6	3	4	12	13^*^
Juggler 15	25	R	6	3	1	13	15^*^

*Handedness was measured by the Edinburgh Handedness Inventory (Oldfield, 1971)*;

*R: right-handed; L: left-handed; RL: no preference*.

* *The jugglers 6, 7, and 8, as well as 9, 10, and 11, as well as 13 and 14 were ranked as equally good*.

### Cinematographic analysis

For a quantitative rating and ranking of the individual juggling expertise, participants were video-taped using a HD-Camcorder placed on a tripod (Panasonic HDC-SD909, CMOS camera systems, Kadoma, Japan) during the entire experimental session. The video was synchronized with the fNIRS system. To enhance the contrast for the cinematographic analysis, the jugglers used one orange ball, which allows the number of loops to be counted, and the remaining balls were white in front of a black carpet (see Figure 1B, Supplementary Video). Videos were taken with a sampling rate of 50 frames per second and a short shutter (1/500 s). For a detailed description of the analyses steps see Section Data Analyses.

### Functional near-infrared spectroscopy (fNIRS)

We recorded hemodynamic responses in sensorimotor (M1, S1, PMC) and visual motion area (V5/MT) on both hemispheres (Hashimoto et al., 2006; Mehnert et al., 2013) using a portable fNIRS system (NIRSport 88 by NIRx Medizintechnik GmbH, Berlin, Germany). fNIRS optode placement was performed using an fNIRS cap (with different sizes) which ensures standardized sensor placement according to the well establishes 10–20 system, which is also used for EEG measurements. The fNIRS setup was similar to Piper et al. (2014) (see Figure 1C) using eight light sources and eight detectors with an inter-optode distance of 2.5–3 cm, which form 16 actual measurement channels. Data was acquired with a sampling frequency of 7.81 Hz.

### Data analyses

#### Quantitative rating and ranking

For quantitative rating, we analyzed the 5-ball cascade only since this task is obviously the most demanding task within our study design for expert jugglers. Here, 10 s time frames within an activation block (5-ball cascade) were rated for the 3rd–6th block of each juggler.

The balls are located in each frame of the video using a Hough Transform (Fernandes and Oliveira, 2012) for circle detection and a limited radius range. Next, associating temporal near ball detections automatically formed the tracks, based on distances in the image plane, color similarity, and temporal distance. During handover, where the hands come to occlude the balls and thereby the circle detection tends to fail, we used a template matching procedure (frame wise comparison) to extend the tracks by the information from previous and later images. For this, an image patch at a track end was used as a template and a similar looking match is searched for within a limited spatial search area around the last detection in the neighboring frame using normed cross correlation. Missing frames were manually tracked. The implementation of the described cinematographic approach uses OpenCV (Bradski, 2000). After this reconstruction we rated the individual trials with three kinematic parameters (Mapelli et al., 2012) to find the level of expertise for each juggler: tosses per second, velocity of the toss, angle of the toss (for left and right hand). For all three parameters, we calculated the average stability, that is, the coefficient of variability independent of the average. The averaged rank of the three behavior measures was calculated. The statistical analysis was performed using SPSS (Version 20.0 for Windows, SPSS Inc., Chicago, IL, USA). Calculating its intra-class correlation (ICC [3,1]) tested the reliability of this procedure with above-mentioned rating of an expert rater. We expect that the quantitative and qualitative rating show comparable ranking positions.

#### Hemodynamics

fNIRS data was analyzed using the software package NIRSlab (v2014.05, NIRx Medical Technologies, New York, United States of America) and Wolfram Mathematica 9.0. Attenuation changes of both wavelengths (850 and 760 nm) were transformed to concentration changes of oxy- and deoxygenated hemoglobin (oxy-Hb and deoxy-Hb, respectively) using the modified Beer-Lambert approach (extinction coefficients: ε_oxyH_b__λ_760_ = 1.4865865 [$\varepsilon_{\text{oxyH}{\text{b}}_{\lambda }850}=2.526391\left[\frac{l}{cm\;mmol}\right],\varepsilon_{\text{deoxyH}{\text{b}}_{\lambda }760}=3.843707\left[\frac{l}{cm\;mmol}\right]$, $\varepsilon_{\text{deoxyH}{\text{b}}_{\lambda }850}=1.798643\left[\frac{l}{cm\;mmol}\right]$; Differential pathlength Factor: 7.25; Cope and Delpy, 1988; Kocsis et al., 2006; Xu et al., 2014). The data was then band-pass filtered to attenuate high-frequency noise and cardiovascular artifacts using 0.01 Hz as high and 0.2 Hz as low pass cutoff frequencies (Huppert et al., 2009). Single trials were baseline corrected (regarding 5 s until stimulus onset). Though the experimental design included long breaks to prevent overlapping of the hemodynamic responses between trials, we directly analyzed the height of amplitude (baseline-corrected average of the temporal window from 5 to 20 s with regard to stimulus onset). Channels were rated as noisy when the first coefficient of the wavelet transform (4th order Daubechies Wavelet) had a higher inter-quartile-range than the 6th one. With this procedure 2.2% of the oxy-Hb and 8.7% of the deoxy-Hb channels were regarded as too noisy and not included in the further analysis (Nakano et al., 2009). Statistical analyses were performed using a one factor (with the six levels: 2-ball, left hand; 2-ball right hand; 3-ball cascade; 5-ball cascade and the two control tasks) repeated measurement ANOVA (within subject design, Greenhouse-Geisser correction; Greenhouse and Geisser, 1959). Partial eta squared of |>0.06| represents a “small” effect size, |>0.01| represents a “medium” effect size and |>0.14| represents a “large” effect size (Cohen, 1992). *Post-hoc* testing included pair-wise comparisons and Bonferroni correction (Dunn, 1961) for the number of tasks. The level of significance was set to 5% (*p* ≤ 0.05) for all tests.

### Ethics committee

There were no minors or persons with disabilities involved in the study and no additional considerations had to be made.

## Results

### Qualitative rating of the level of expertise

The expertise level between jugglers differed individually (see Table 1), especially for the maximum number of reliably handled balls, which fits well with previous studies investigating expert jugglers such as Gerber et al. (2014). Quantitative results provided by an expert rater (also an experienced juggler) are shown in Table 1 as well.

### Quantitative rating of the level of expertise

During the execution of the 5-ball cascade, three jugglers had no failure, two jugglers had one failures (dropping balls), five juggler had two failures, two jugglers had three failures and three jugglers had four failures. Finally, we collected eight valid trials for each participant. The cinematographic analysis revealed between 128 and 163 tosses for each participant (see Table 2). The analysis of the tosses per seconds resulted in an inter-subject average frequency of 4.1 ± 0.3 Hz. Average velocities at the toss were 7.6 ± 0.6 ms^−1^ (left hand) and 7.4 ± 0.6 ms^−1^ (right hand), and the average angles 83.0 ± 1.1° (left hand) and 83.2 ± 1.3° (right hand), respectively.

**Table 2:** **Cinematographic data, Expertise-Rating (rank)**.

**VP**	***n***	**Tosses per second [Hz]**			**Release speed [ms**^**−1**^**]**						**Release angle Φ [°]**						**Ørank^b^**
					**Left hand**			**Right hand**			**Left hand**			**Right hand**			
		***M***	***95% CI***	***CV% ^a^***	***M***	***95% CI***	***CV%***	***M***	***95% CI***	***CV%***	***M***	***95% CI***	***CV%***	***M***	***95% CI***	***CV%***	
Juggler 1	148	3.99	[3.95 4.04]	6.75	7.63	[7.59 7.66]	1.97	7.39	[7.35 7.43]	2.30	83.04	[82.84 83.23]	1.06	83.42	[83.21 83.62]	1.08	1
Juggler 2	163	4.37	[4.32 4.43]	8.23	6.94	[6.90 6.98]	2.45	6.66	[6.62 6.70]	2.70	82.70	[82.54 82.87]	0.92	81.63	[81.43 81.84]	1.15	2
Juggler 3	155	4.16	[4.11 4.21]	7.68	7.27	[7.23 7.32]	2.48	6.89	[6.85 6.94]	2.90	83.77	[83.51 84.00]	1.30	82.48	[82.18 82.73]	1.50	3
Juggler 4	143	3.91	[3.84 3.97]	10.29	7.49	[7.45 7.56]	3.20	7.38	[7.32 7.45]	3.66	82.69	[82.48 82.85]	0.96	83.26	[82.99 83.47]	1.24	5
Juggler 5	150	4.07	[3.99 4.15]	12.04	7.69	[7.64 7.76]	3.64	7.79	[7.71 7.87]	4.75	82.80	[82.57 83.06]	1.33	82.68	[82.40 82.95]	1.46	13
Juggler 6	150	4.07	[3.98 4.15]	12.87	7.92	[7.87 7.96]	2.65	7.49	[7.45 7.54]	2.54	84.21	[84.00 84.38]	1.00	83.03	[82.75 83.28]	1.42	4
Juggler 7	162	4.36	[4.29 4.42]	8.98	7.25	[7.20 7.30]	3.03	6.80	[6.75 6.84]	3.24	82.87	[82.62 83.14]	1.41	81.04	[80.79 81.34]	1.55	7
Juggler 8	160	4.31	[4.23 4.39]	11.79	7.00	[6.95 7.04]	3.00	7.18	[7.14 7.22]	2.79	83.20	[82.94 83.45]	1.43	84.50	[84.28 84.74]	1.23	6
Juggler 9	147	3.99	[3.92 4.06]	10.54	7.18	[7.11 7.23]	3.76	7.47	[7.42 7.53]	3.21	82.17	[81.87 82.46]	1.59	84.25	[83.96 84.51]	1.42	10
Juggler 10	156	4.24	[4.17 4.32]	11.33	6.94	[6.90 6.99]	2.88	7.10	[6.96 7.22]	8.45	82.75	[82.49 83.02]	1.45	83.25	[83.04 83.45]	1.14	8
Juggler 11	185	4.97	[4.90 5.04]	10.27	7.49	[7.44 7.53]	2.94	6.79	[6.73 6.85]	4.27	79.85	[79.54 80.16]	1.89	82.35	[82.03 82.67]	1.91	12
Juggler 12	155	4.17	[4.10 4.24]	10.05	7.48	[7.40 7.55]	4.41	7.35	[7.29 7.40]	3.40	82.70	[82.27 83.06]	2.13	81.75	[81.31 82.21]	2.42	15
Juggler 13	150	4.10	[4.00 4.19]	14.57	8.12	[8.04 8.21]	4.80	7.93	[7.86 8.01]	4.29	84.28	[83.95 84.50]	1.40	84.36	[84.07 84.60]	1.38	14
Juggler 14	128	3.44	[3.40 3.48]	7.05	9.18	[9.10 9.25]	3.27	9.07	[9.01 9.12]	3.75	83.54	[83.19 83.91]	1.74	83.83	[83.54 84.12]	1.41	8
Juggler 15	141	3.90	[3.79 4.02]	17.90	7.64	[7.56 7.71]	4.19	7.86	[7.80 7.95]	4.07	84.62	[84.39 84.82]	1.08	85.93	[85.68 86.21]	1.28	11

a *Coefficient of variation*;

b *motoric expertise: average ranking position of the coefficient of variation*;

*n, number of analyzed loops*.

The ranking carried out by experts and the quantitative analysis of the stability measures shows a good fit [inter-rater-reliability: *ICC*(3,1), *absolute* = 0.763; *p* = 0.001]. There are no significant correlations between the level of juggling expertise and the number of tosses per second [*r*_*s*__(15)_ = –0.004, *p* = 0.990], the release speed [left hand: *r*_*s*__(15)_ = 0.282, *p* = 0.309; right hand: *r*_*s*__(15)_ = 0.350, *p* = 0.201] und the release angles [left hand: *r*_*s*__(15)_ = –0.097, *p* = 0.732; right hand: *r*_*s*__(15)_ = 0.116, *p* = 0.680].

### Subjective rating of the complexity of tasks

Expert jugglers rated the control tasks as the ones with lowest complexity, followed by the 3-ball cascade, 2-ball juggling with the left hand, 2-ball juggling with the right hand, and the 5- ball cascade [Friedman-Test: $X_{\left(2,\;30\right)}^{2}$ = 36.66, *p* < 0.001, Table 3].

**Table 3:** **Task complexity and hemodynamic responses (grand averages) during control and juggling tasks, rmANOVA**.

	**(1) Control motion 1 Hz**		**(2) Control motion 2 Hz**		**(3) 3-ball cascade**		**(4) 2 balls right hand**		**(5) 2 balls left hand**		**(6) 5-ball cascade**		***F***	***p*^a^**	**η^2^**	***Post-hoc* Bonferroni^b^**
	***M***	***95% CI***	***M***	***95% CI***	***M***	***95% CI***	***M***	***95% CI***	***M***	***95% CI***	***M***	***95% CI***				
**TASK COMPLEXITY**																
	1.00	[1.00 1.00]	1.00	[1.00 1.00]	1.60	[1.28 1.92]	2.07	[1.54 2.59]	2.53	[2.11 2.96]	6.53	[5.85 7.22]	3	<0.001	−	1,2 < 3 < 4 < 5 < 6
**HEMODYNAMIC RESPONSES (OXY-Hb)**																
PMC_*L*_	0.37	[0.12 0.63]	0.48	[0.20 0.76]	0.66	[0.35 0.97]	0.98	[0.64 1.32]	1.05	[0.72 1.38]	2.04	[1.45 2.63]	20.23	<0.001	0.65	1 < 4,5 < 6
M1_*L*_	0.54	[0.36 0.73]	0.70	[0.47 0.94]	0.94	[0.61 1.26]	1.59	[1.15 2.03]	1.61	[1.24 1.98]	3.69	[3.04 4.34]	55.98	<0.001	0.84	1,2 < 4,5 < 6
S1_*L*_	0.56	[0.32 0.80]	0.84	[0.56 1.11]	0.89	[0.50 1.29]	1.76	[1.25 2.27]	2.08	[1.34 2.82]	4.37	[3.48 5.27]	37.59	<0.001	0.77	1,2,3 < 4,5 < 6
MT/V5_*L*_	0.47	[0.20 0.74]	0.71	[0.28 1.14]	0.84	[0.48 1.20]	1.20	[0.70 1.71]	1.49	[1.06 1.92]	3.89	[2.97 4.81]	47.92	<0.001	0.81	2,3,4 < 6
PMC_*R*_	0.44	[0.15 0.73]	0.56	[0.27 0.86]	0.70	[0.38 1.02]	1.18	[0.75 1.62]	1.10	[0.70 1.51]	3.14	[1.91 4.37]	21.39	<0.001	0.66	1,2,3 < 4,5 < 6
M1_*R*_	0.70	[0.22 1.17]	0.84	[0.48 1.20]	1.05	[0.53 1.56]	1.59	[1.13 2.06]	1.70	[1.17 2.23]	4.25	[2.70 5.80]	16.39	<0.001	0.60	1,2,3,4 < 6
S1L_*R*_	0.53	[0.25 0.81]	0.65	[0.31 1.00]	0.90	[0.47 1.34]	1.78	[1.15 2.41]	1.46	[1.05 1.87]	4.55	[3.38 5.73]	41.71	<0.001	0.81	1,2,3 < 4,5 < 6
MT/V5_*R*_	0.47	[0.12 0.83]	0.61	[0.18 1.04]	0.78	[0.38 1.17]	1.66	[1.09 2.24]	1.00	[0.51 1.50]	3.48	[2.38 4.58]	28.87	<0.001	0.72	1,2,3 < 4,5 < 6
**HEMODYNAMIC RESPONSES (DEOXY-Hb)**																
PMC_*L*_	−0.03	[−0.06 0.01]	−0.04	[−0.08 0.00]	−0.07	[−0.11 -0.03]	−0.11	[−0.19 -0.02]	−0.03	[−0.09 0.03]	−0.02	[−0.21 0.18]	0.92	0.381	0.08	ns^c^
M1_*L*_	−0.12	[−0.19 -0.05]	−0.12	[−0.19 -0.05]	−0.16	[−0.25 -0.08]	−0.15	[−0.24 -0.06]	−0.01	[−0.08 0.07]	0.20	[−0.06 0.45]	9.93	0.005	0.47	1,3,4 < 5
S1_*L*_	−0.06	[−0.06 0.07]	−0.06	[−0.06 0.10]	−0.09	[−0.09 0.14]	−0.05	[−0.05 0.20]	0.09	[0.09 0.15]	0.19	[0.19 0.43]	4.12	0.048	0.34	ns
MT/V5_*L*_	−0.04	[−0.06 -0.01]	−0.02	[−0.05 0.01]	−0.05	[−0.08 -0.02]	−0.07	[−0.13 -0.02]	0.00	[−0.07 0.06]	0.02	[−0.11 0.15]	1.94	0.173	0.15	ns
PMC_*R*_	−0.07	[−0.10 -0.03]	−0.07	[−0.11 -0.03]	−0.11	[−0.18 -0.04]	−0.02	[−0.16 0.11]	−0.05	[−0.19 0.10]	0.25	[−0.18 0.68]	2.54	0.128	0.19	ns
M1_*R*_	−0.11	[−0.19 -0.04]	−0.15	[−0.25 -0.05]	−0.22	[−0.36 -0.08]	−0.08	[−0.29 0.13]	−0.15	[−0.31 0.00]	0.11	[−0.40 0.62]	1.60	0.233	0.13	ns
S1L_*R*_	0.00	[−0.41 0.41]	0.04	[−0.92 1.00]	−0.04	[−0.44 0.35]	0.07	[−1.04 1.18]	0.04	[−1.31 1.39]		−^d^	–	–	–	ns
MT/V5_*R*_	−0.02	[−0.05 0.02]	−0.01	[−0.06 0.03]	−0.05	[−0.09 0.00]	0.00	[−0.08 0.09]	−0.04	[−0.10 0.02]	0.02	[−0.22 0.26]	0.34	0.621	0.03	ns

a Greenhouse-Geisser correction;

b pairwise comparisons (number of control and juggling tasks, only significant results);

c not significant;

d *too much noisy data, M, mean value; SE, standard error; L/R, left/right; PMC, premotor area; M1, primary motor area; S1, primary somatosensory area; MT/V5, visual area 5*.

### Hemodynamics

#### Task related differences

**Figure 2:**
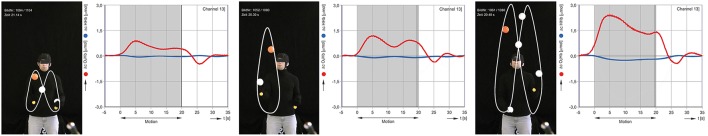
**Exemplary results for cinematographic, semi-automated tracking of the balls during juggling**. Concentration changes of oxygenated (red) and deoxygenated (blue) hemoglobin in 2-, 3- and 5-ball juggling (average of 8 trials of juggler 2). The gray area marks the onset and duration of the juggling activity.

**Figure 3:**
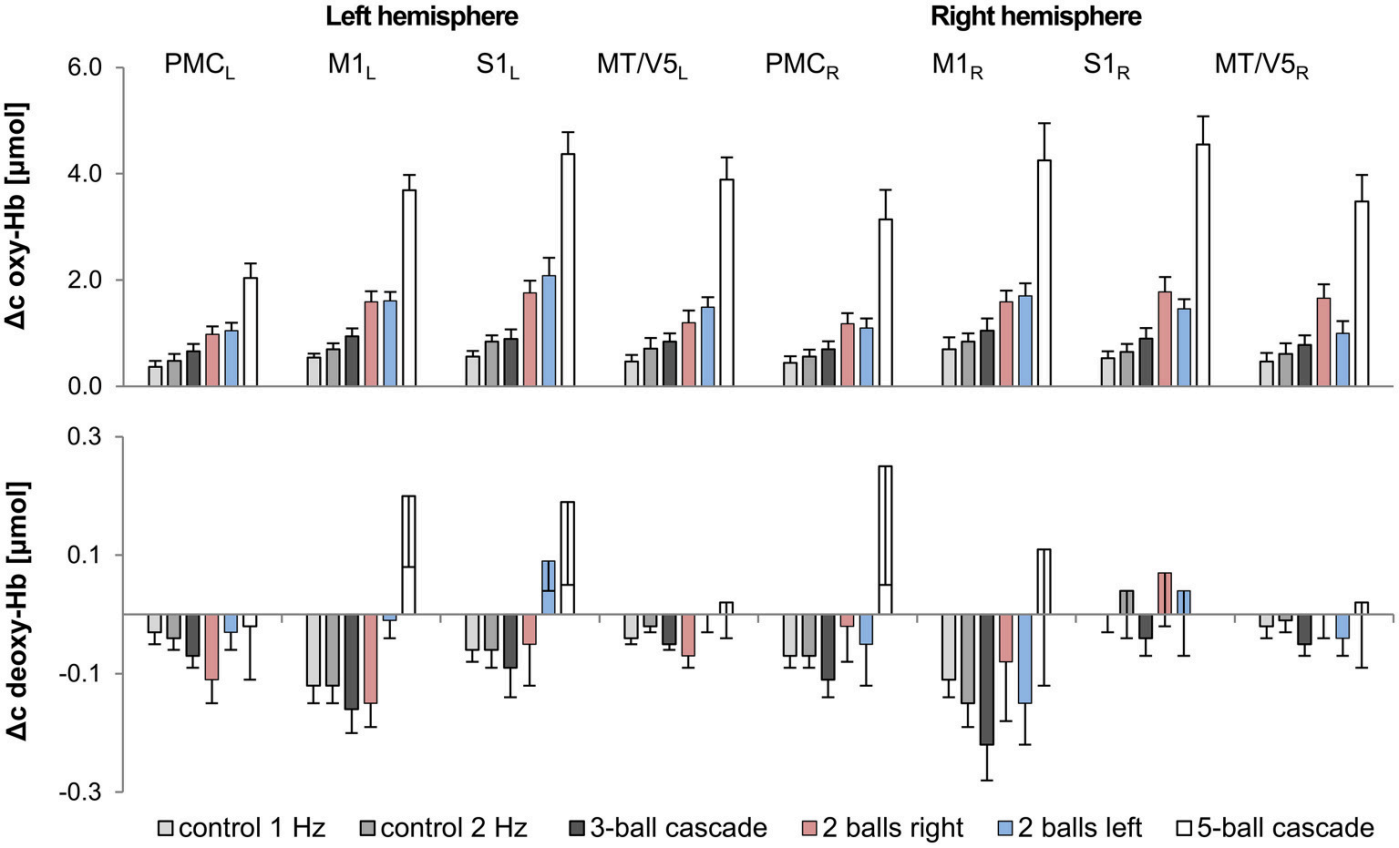
**Comparison of hemodynamic responses** [oxy-Hb (upper chart) and deoxy-Hb (lower chart); mean across subjects; error bars, Standard Error of the Mean] among the 8 ROIs during control and juggling tasks, L/R, left/right; PMC, premotor area; M1, primary motor area; S1, primary somatosensory area; MT/V5, visual area 5.

A repeated measures ANOVA with factor TASK (3- and 5-ball cascade, one handed 2-ball juggling [left and right hand], 1 and 2 Hz control tasks) revealed a significant increase in oxy-Hb as a function of task complexity for bilateral M1, S1, PMC and MT/V5 (for details see Figures 2, 3 and Table 3). More specifically, those tasks that were ranked as less complex were those that show smallest hemodynamic responses in oxy-Hb and vice versa. On the other hand, no such effects were observed for deoxy-Hb, except for left M1 (see Table 3).

### Differences related to individual level of expertise

**Figure 4:**
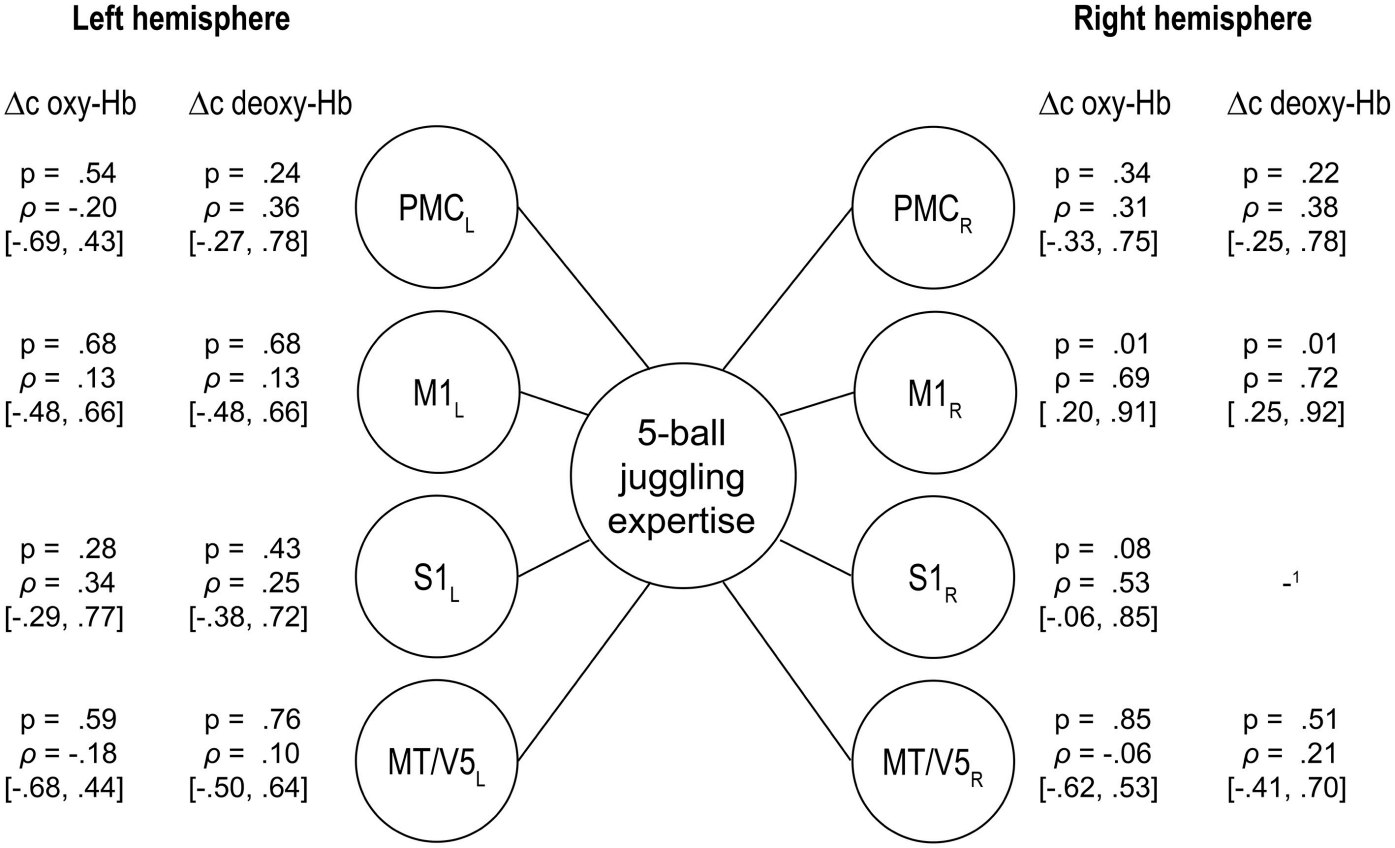
**Spearmann's rank correlations between the individual level of expertise as rated by the cinematographic video analysis of the 5-ball cascade and the changes in oxy- and deoxygenated hemoglobin (95% confidence intervals in brackets, uncorrected for multiple comparisons), PMC, premotor area; M1, primary motor area; S1, primary somatosensory area; MT/V5, visual area 5**. The results in right M1 confirm: the higher the expertise, the lower the hemodynamic response (^1^too much noisy data).

For the 5-ball cascade, the level of expertise did not correlate with the height of oxy- nor deoxy-Hb within M1, S1, PMC, and MT/V5 when correcting for multiple comparisons. Furthermore, we did a partial correlation analysis to control for the effect of movement speed. The results of the correlations analysis remained non-significant [Δc oxy-Hb: *r*_(9)_ = −0.37 − 0.43, *p* = 0.18 − 0.88; Δc deoxy-Hb: *r*_(9)_ = −0.01 − 0.74, *p* = 0.15 − 0.99). However, there was a trend within right M1 toward a negative association between the hemodynamic response and the level of juggling expertise (oxy-Hb: M1_R_ ρ = 0.694, *p* = 0.012; deoxy-Hb: M1_R_ ρ = 0.718, *p* = 0.009; uncorrected for multiple comparisons, see Figure 4). More specifically, higher level of expertise tends toward lower hemodynamic responses within right M1 only.

## Discussion

Based on our findings, we confirmed that fNIRS is a capable tool to investigate online brain processes during the execution of certain motor tasks. Here, we extended previous findings by showing that the execution of a complex, sports-related motor task such as juggling is associated with neurovascular changes in sensorimotor (M1, S1, PMC) as well as visual motion areas (MT/V5). The novelty of the present study is that those brain regions seem to be modulated by task complexity, since a 5-ball cascade showed enhanced hemodynamic responses for oxy-Hb as compared to less complex tasks such as a 3- or 2- ball juggling pattern. However, in contrast to previous findings (Gerber et al., 2014) that showed an association between gray matter density and the level of juggling expertise in MT/V5, we here found only non-significant trend in M1 on a functional level.

### Quantitative rating of the level of expertise

The small inter-individual differences in the main tossing parameters *release angle* and *release speed* during 5-ball juggling confirms the expert level in our study population. However, the cinematographic analyses revealed individual differences in the variability of the parameters and thereby differences in the level of expertise between the jugglers. As the quantitative analyses reports similar ranking positions as compared to the subjective rating by an expert, our results emphasize the validity of the quantitative approach.

### Hemodynamics

For the first time, according to our knowledge, we were able to show the adaptability of the human brain as a consequence of task complexity during the execution of a sports-related motor task. Subjectively rated levels of complexity for the different tasks are associated with the amount of hemodynamic response [oxy-Hb, for all areas *p* < 0.001; η^2^ = 0.60 − 0.84] in sensorimotor and visual motion areas. So far, studies have only reported that higher movement speed when walking and running (Suzuki et al., 2004; Harada et al., 2009) is associated with higher hemodynamic responses (oxy-Hb). We extended these findings by showing that the hemodynamic response seems not only be affected by movement speed since control experiments (2 Hz alternating arm movements) indicated that the hemodynamic response was lower compared to 2-ball juggling. Furthermore, the hemodynamic response of the 3-ball cascade (~3 Hz movements speed) was lower than 2-ball juggling (~2 Hz movement speed).

In contrast, with regard to deoxy-Hb, the majority of subjects in the present study did not show any significant alteration during the execution of ball juggling with different degrees of complexity. The detailed underlying mechanisms for the lack of effects on deoxy-Hb remain unknown and cannot be answered with our study design. However, it is tempting to speculate that the amplitude for deoxy-Hb in general is quite small and or less prone to alterations (induced by task complexity) as compared to oxy-Hb. It is interesting to see, however, that there was an “atypical” increase in deoxy-Hb during 5-ball juggling in the majority of jugglers (see Figure 2 and Table 3). The typical hemodynamic response to motor stimulation would include a reduction in deoxy-Hb concentration. The reason for this response is an increased regional blood flow (rCBF) in order to meet the higher demand in oxygen and thus higher “washout” of deoxy-Hb. In contrast, 5-ball juggling potentially causes a higher extraction of oxygen. This high cerebral metabolic rate of oxygen consumption (CMRO2) compensates the washout effect.

During the execution of 5-ball juggling we observed a trend toward a negative relationship between the 5-ball juggling expertise and alterations in oxy- and deoxy-Hb for right M1. This effect did not reach significance potentially due to the relative small number of participants included in the present study. However, even that trend is in line with previous studies investigating structural correlates of juggling expertise (Gerber et al., 2014). Similar findings of such brain-behavior relationships when learning to perform complex motor tasks have been shown on a structural as well as functional level (Taubert et al., 2010, 2011; Gryga et al., 2012).

### Limitations

Within each of the analyzed juggling and control tasks hemodynamic responses (oxy-Hb) in sensorimotor areas as well as in visual motion area MT/V5 were quite similar (see Table 3 and Figure 2). Hence, it is reasonable to assume that hemodynamic changes in oxy-Hb are not task related but due to a global effect caused by alterations in extra-cerebral blood flow (Kirilina et al., 2012). However, we believe that our findings are potentially not due to an increase in extra-cerebral blood flow, since we were able to show regional specific effects for deoxy-Hb. Deoxy-Hb is consistently less sensitive to alterations in extra-cerebral blood flow (Piper et al., 2014). Furthermore, the execution of unimanual 2-ball juggling in the left hand revealed higher neurovascular changes (see Table 3 and Figure 2, deoxy-Hb) in primary motor cortex on the contralateral hemisphere as compared to the ipsilateral hemisphere [*t*_(14)_ = 3.19, *p* < 0. 01]. Testing the same difference during unimanual 2-ball juggling in the right hand fails to attain statistical significance [*t*_(14)_ = –1.23, *p* = 0.24].

Since task complexity during juggling is associated with higher movement speed, which may influence hemodynamic responses (Obrig et al., 1996), we performed two control experiments. Here, we were able to show, that hemodynamic responses during the execution of 2-ball juggling compared to a 2 Hz control task, which involves a similar movement frequency, were higher. We interpret this finding as a task complexity-dependent effect. Another potential limitation of the present study is that we only controlled for movement frequency for the 2-ball juggling task and not for 3- and 5-ball juggling, which obviously has to be performed with higher movement speed up to 4 Hz for 5-ball juggling. Hence, we cannot totally rule out the possibility that the observed effects (modulation of oxy-Hb with increasing complexity) rely on movement frequency.

Additionally, comparisons in our study design were made between uni- (2-ball juggling) and bimanual juggling tasks (3- and 5-ball cascade), a factor that might influence the interpretation of the observed effects as well. More specifically, one could argue that the observed effects are not related to task complexity but displays a simple difference between uni- and bilateral tasks. However, this is unlikely since 3-ball cascades showed less oxy-Hb alterations as compared to 2 ball (unilateral) juggling.

## Author contributions

DC and CA: Conceived and designed the experiments; DC and CA: Performed the experiments; DC, MC, MB, and JM: Analyzed the data; MC and MB: Contributed materials/analysis tools; DC, PR, and JM: Interpretation of data for the work.

### Conflict of interest statement

The authors declare that the research was conducted in the absence of any commercial or financial relationships that could be construed as a potential conflict of interest.
